# On-Surface Ullmann-Type Coupling Reactions of Aryl Halide Precursors with Multiple Substituted Sites

**DOI:** 10.3390/nano15090646

**Published:** 2025-04-24

**Authors:** Qiwei Liu, Yuhong Gao, Chi Zhang

**Affiliations:** Interdisciplinary Materials Research Center, School of Materials Science and Engineering, Tongji University, Shanghai 201804, China

**Keywords:** on-surface synthesis, Ullmann-type coupling, low-dimensional nanostructure, substituted site, scanning probe microscopy

## Abstract

The fabrication of low-dimensional nanostructures through on-surface synthesis has emerged as a promising strategy for developing high-precision electronic devices. Among various reactions, Ullmann-type coupling (with carbon–halogen bond activation) stands out in this field as a prevalent methodology due to its straightforward activation process, highly programmable characteristics, and remarkable synthetic efficiency. To date, on-surface Ullmann-type coupling reactions of aryl halide precursors have been extensively studied with the assistance of in situ characterization techniques. The resulting carbon-based nanostructures exhibit high structural diversity and significant potential for applications in molecular electronics. This review categorizes recent progress in the precise preparation of carbon-based nanostructures based on molecular precursors with distinct halogen substituted sites, including *para*-, *meta*-, and *ortho*-sites, *peri*- and *bay*-regions, and their combination. In addition, systematic analysis and comparative discussion of their respective characteristics is also provided.

## 1. Introduction

In 1975, Gordon E. Moore proposed the well-known law that the number of transistors on a microchip would double approximately every two years [1]. The law has been invalidated because the advancement of manufacturing processes is approaching its physical limits, which hinders the further development of microelectronic devices [2]. Low-dimensional carbon-based nanomaterials, such as graphene nanoribbons (GNRs) and nanographenes (NGs), have demonstrated excellent electronic properties and are believed to be the potential alternatives to conventional silicon-based materials in the fabrication of next-generation nanoscale electronic devices [3,4]. Particularly, π-conjugated nanomaterials are promising for fabricating graphene-nanoribbon field-effect transistors [5] and for molecular switching [6]. Nonetheless, these applications require atomically precise fabrication of large-scale carbon nanostructures on substrates, which poses a great challenge in the preparation of these materials. The advancement of scanning probe microscopy (SPM) techniques over the past few decades, such as ultra-high vacuum scanning tunneling microscopy (UHV-STM) and non-contact atomic force microscopy (nc-AFM) [7,8], has enabled the real-space visualization of molecule-based nanostructures through on-surface reactions [9,10,11]. In addition, theoretical calculations, such as density functional theory (DFT) calculations and molecular dynamics (MD) simulations, have been applied to verify the product structures, elucidate reaction mechanisms [12], simulate reaction processes [13,14], etc. Consequently, the pioneering study by Grill et al. established the bottom-up on-surface synthesis strategy utilizing the self-assembly and polymerization of molecular precursors on well-defined surfaces [15], which has opened new avenues for the precise fabrication of carbon-based nanostructures on metal substrates [16,17].

**Scheme 1:**
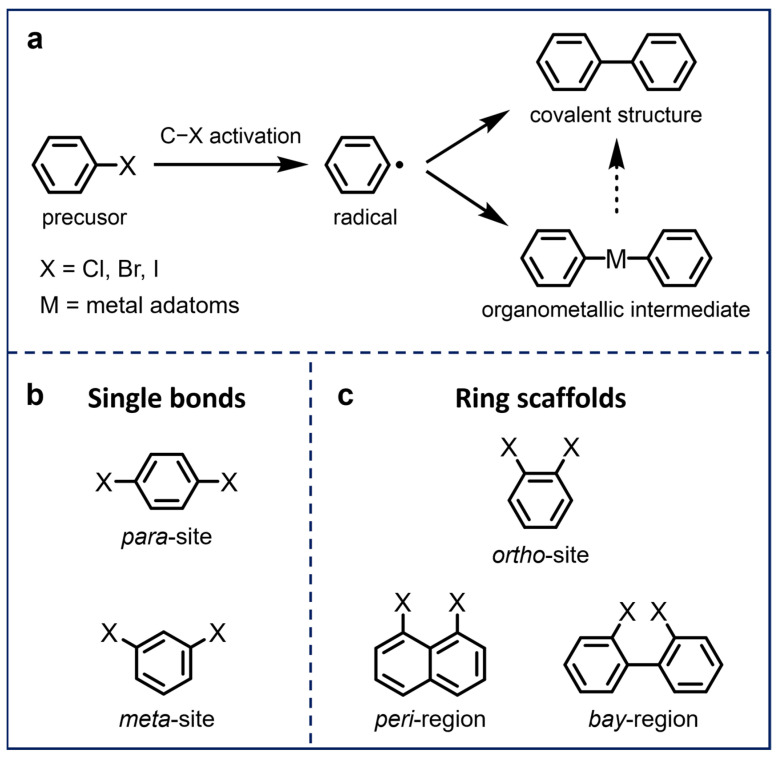
(**a**) Illustration of the on-surface Ullmann-type coupling reaction of aryl halides. (**b**,**c**) Typical halogen-substituted sites of aryl halide precursors, which generally form (**b**) single bonds and (**c**) ring scaffolds after on-surface Ullmann-type coupling.

In order to fabricate functional low-dimensional π-conjugated nanostructures that satisfy the aforementioned requirements, efficient on-surface reactions have been adopted to create specific C–C conjunctions between molecules. The Ullmann coupling reaction, a classic method for the preparation of complex aryl derivatives in solution chemistry [18], has also been extensively used in on-surface synthesis since the seminal work by Grill et al. [15], owing to its highly tunable reaction conditions and predesigned reaction manners [19]. This on-surface Ullmann-type coupling reaction can be initiated by activating molecular precursors through thermal annealing [20], SPM tip manipulation [21,22], and light illumination [23]. As illustrated in Scheme 1a, the reaction generally starts with the dehalogenation of aryl halide precursors to form surface-stabilized radicals. These activated radicals subsequently undergo one of the two following potential reaction pathways, depending on the surface properties. The first pathway involves direct radical polymerization, leading to the formation of covalent bonds, while the second pathway involves the combination of radicals with metal adatoms to form organometallic (OM) intermediates and the removal of the adatoms via further annealing to form covalent structures [24,25] (which depends on the strength of the OM C–M–C bonds).

A considerable number of studies have been conducted on the precise synthesis of carbon-based nanostructures via on-surface Ullmann-type coupling reactions. The resulting nanostructures range from zero-dimensional (0D), one-dimensional (1D), to two-dimensional (2D), based on the functionalization of aryl halide precursors, in particular, the functionalized sites and numbers of halogen substituents. Conventionally, molecular precursors are functionalized with single halogen substitutions [20,26,27,28,29] or multiple halogen substitutions at *para*-sites [30,31,32] and *meta*-sites [33,34,35] (Scheme 1b), leading to the formation of C–C single bonds exclusively after dehalogenative coupling processes. As an extension, (poly)halogenated molecules with halogens substituting different adjacent sites, typically including *ortho*-sites [36,37,38,39,40], *peri*-regions [41,42], and *bay*-regions [43,44,45,46,47,48,49], can further form various structures due to their unique spatial configurations and distinct activation barriers, which expands the functionality of traditional Ullmann coupling and allows for the direct construction of ring scaffolds for more complex nanostructures (Scheme 1c) [50]. In this review, we summarize recent research works on the on-surface synthesis of carbon-based nanostructures using aryl halide precursors with multiple substituted sites on metal substrates and characterized by UHV-SPM, which are classified into five different categories, as displayed in Scheme 1. Several representative resulting nanostructures and corresponding reaction pathways and mechanisms are also discussed.

## 2. Precursors with *Para*-Site Substitutions

Precursors with *para*-site halogen substitution are commonly used in early studies. Due to the configuration of such halogen substitutions, these precursors typically form 1D chain structures via Ullmann-type coupling.

**Figure 1:**
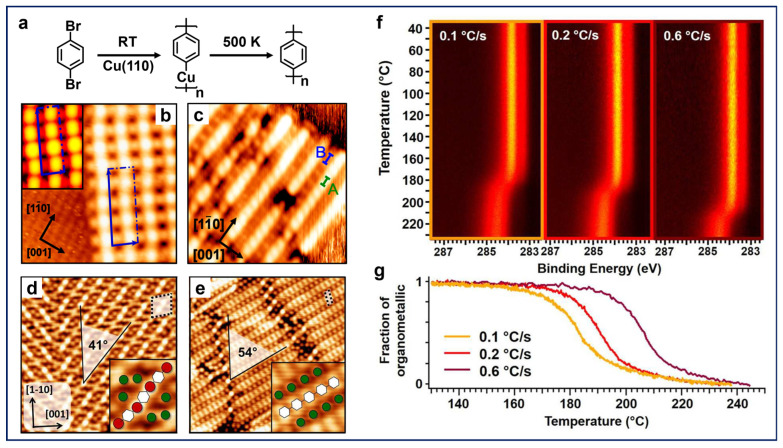
(**a**) Scheme for the polymerization of dBB molecules on Cu(110). (**b**) STM image of the OM intermediates on Cu(110) formed by the deposition of dBB at room temperature (RT). Inset: STM simulation of the structure. (**c**) Covalent chains on Cu(110) obtained after annealing the sample at 500 K. Reprinted with permission from Ref. [30]. (**d**) OM intermediates and (**e**) polymeric chains with different orientations at a higher molecular coverage than (**b**,**c**). The insets illustrate the detailed structure of the two phases in which white hexagons and red and green circles indicate phenylene units and Cu and Br atoms, respectively. (**f**) Fast-XPS maps of the C 1s signals obtained during the coupling process of dBB on Cu(110) under different annealing rates. (**g**) Kinetic curves representing the changes in the fraction of OM intermediates with respect to different annealing temperatures for different heating rates. Reprinted with permission from Ref. [51].

Contini et al. presented a systematic study of the carbon–halogen (C–X) activation of *para*-halogenated benzene molecules on metal surfaces [30,51,52]. The polymerization of the 1,4-dibromobenzene (dBB) molecules on a Cu(110) surface is illustrated in Figure 1a. Due to the high reactivity of copper surfaces, OM intermediates are generally involved in the Ullmann-type reaction processes [25,26], featuring bright dots (Cu adatoms in this case) between neighboring molecular components in the chains (Figure 1b). Subsequently, high annealing temperatures (500 K) induce the removal of Cu adatoms along with the transformation from OM chains to more uniform polymeric chains, with the absence of bright dots in Figure 1c, after annealing, which is a distinctive feature of covalent bonds [30]. Remarkably, OM intermediates and polymeric chains were found to have different orientations on the surface with respect to the close-packed [1$\overset{-}{1}$0] direction (Figure 1d,e), which can be attributed to the difference in periodicity between the two structures [51], also implying the diffusion of molecules during the transition. Furthermore, the role of kinetic factors on the reaction temperature at which covalent chains are generated from OM intermediates has also been investigated, considering that the on-surface reactions are usually controlled by a combination of thermodynamic and kinetic factors. The fast X-ray photoelectron spectroscopy (fast-XPS) maps for the C 1s signals and kinetic curves demonstrated that varying the heating rate resulted in a shift in the onset of the reaction temperature of the conversion from the OM intermediates to covalent chains (Figure 1f,g). It is noteworthy that a faster heating rate led to a higher onset temperature, indicating a nonequilibrium transition. This can be attributed to the diffusion-controlled polymerization process, where a slower heating rate permits a higher diffusion opportunity with longer reaction time (kinetics) for the reactants on the surface [51]. Such kinetic measurements experimentally reveal the evolution from OM structures to covalent bonding and directly provide mechanistic insights into the reaction pathways.

In addition, the role of different types of halogens is often considered for halogenated precursors [20,53,54], which usually leads to different C–X activation temperatures and barriers [55]. For example, on-surface Ullmann-type reactions with *para*-halogenated benzene molecules (with the same backbone as described in Figure 1), involving chlorine (Cl), bromine (Br), and iodine (I) substitutes on either site, have been systematically explored by the same group [56]. The findings conclude that the corresponding C–X bonds have disparate dissociation temperatures (Cl > Br > I) and exhibit the process of sequential dehalogenation. The varying dehalogenation temperature windows provide valuable possibilities for the precursor design as the introduction of different halogen substituents allows temperature-dependent hierarchical reactions to occur in a predetermined sequence and manner [57].

In general, the on-surface Ullmann coupling reaction of *para*-halogenated precursors follows the sequence of the OM chain formation and the removal of adatoms to form covalent chains, leading to relatively simple structures [30,32,58]. Furthermore, the reaction can be modulated by several experimental conditions, including kinetic factors (heating rate) [51], molecule coverage [32,59], the existence of halogens [56,60], and the activity of metal substrates [31]. These findings have contributed to a deeper understanding of the underlying mechanisms of on-surface Ullmann dehalogenative coupling reactions.

## 3. Precursors with *Meta*-Site Substitutions

In addition to the *para*-site halogenated molecules, precursors with *meta*-site halogen substituents are also capable of forming single bonds via on-surface Ullmann coupling. It generally leads to the construction of 2D carbon-based nanostructures [33,34,61] and expands the diversity of on-surface synthesized nanomaterials.

**Figure 2:**
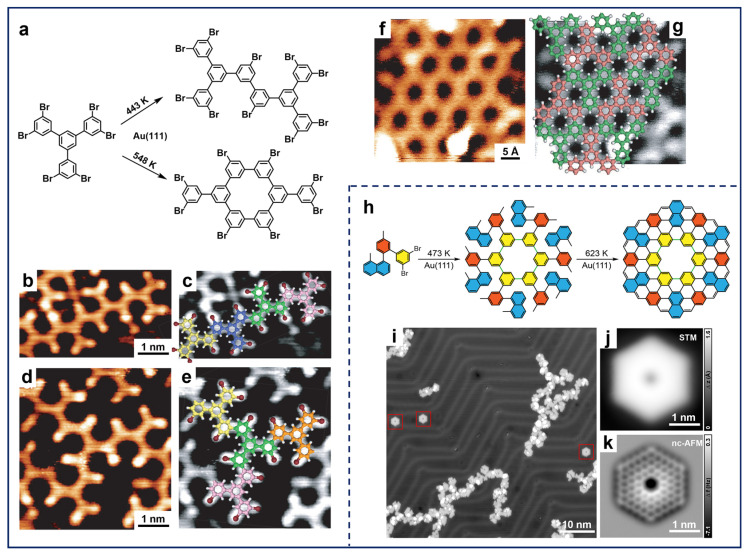
(**a**) Scheme for the Ullmann coupling of TDBPB molecules on Au(111). (**b**–**e**) STM images of single-covalent-bonded nanostructures on Au(111) obtained after annealing at 443 K, superimposed with corresponding structural models. (**f**) STM image of the porous covalent nanoarchitecture obtained after annealing at 548 K with (**g**) corresponding structural model. Reprinted with permission from Ref. [65]. (**h**) Scheme for on-surface synthesis of C144 hexagonal coronoid on Au(111). (**i**) Large-scale STM image obtained after the final step of annealing at 623 K. The target products are marked by the red squares. (**j**) STM and (**k**) nc-AFM images of C144 hexagonal coronoid. Reprinted with permission from Ref. [35].

One prevailing focus of studies on *meta*-halogenated molecules has been the construction of polybiphenyl rings and 2D porous network structures [33,62]. Due to the distinct energy barriers of different pathways during the whole reaction process and the unique features of metal substrates, the configuration of intermediates and final products can be varied, including *cis-trans* isomerism and different chirality [63,64,65]. Additionally, the extent of dehalogenation is found to be correlated with temperatures, which leads to the discrepancy in the formation of different linkages. Accordingly, Silly et al. showed that the number of intermolecular covalent bonds can be modulated by controlling the annealing temperature, which altered the selectivity of the products (Figure 2a) [65]. After the deposition of 1,3,5-tris(3,5-dibromophenyl)benzene (TDBPB) molecules onto Au(111) held at RT followed by annealing at 443 K, one of the C–Br bonds at the *meta*-site dissociated and covalent structures were formed (Figure 2b,d). Superimposed chemical models (Figure 2c,e) exhibited that a single-covalent-bond linkage of the *meta*-site was formed between the adjacent monomers. In contrast, annealing the sample at 548 K after deposition caused more complete dissociation of C–Br bonds at each *meta*-site, leading to the formation of different covalent building blocks (Figure 2a) and an interconnected porous network structure in an extended manner (Figure 2f,g). This process clearly indicates the temperature-dependent on-surface hierarchical engineering of porous graphene nanoarchitectures. In addition, in the case of other substrates such as Ag(111) and Cu(111), OM intermediates are inevitably involved in the fabrication of corresponding nanostructures prior to the formation of covalent bonds [62,63,66,67].

Additionally, the construction of nanostructures using *meta*-halogenated precursors is not solely limited to on-surface Ullmann-type coupling. The delicate design of molecular precursors can also facilitate further carbon–hydrogen (C–H) activation, which enables the construction of more sophisticated nanostructures [68,69]. For example, Yu et al. reported the on-surface synthesis of zigzag coronoid C144 (Figure 2h) based on a well-designed precursor consisting of 3,5-dibromobenzene [35]. The precursors initially formed single-bonded ring structures via an on-surface Ullmann-type reaction after complete dehalogenation at the *meta*-sites. Subsequently, further annealing at 623 K resulted in cyclohydrogenation forming a C144 hexagonal coronoid (Figure 2i). The chemical structure was further unambiguously characterized by STM and nc-AFM showing zigzag outer edges (Figure 2j,k). Interestingly, Gottfried et al. demonstrated that halogen substitution at the *meta*-site also reduced the activation barrier of the central C–H bond between the two substituents, providing the potential for additional structural cyclization [70]. Notably, due to the generality of such a reaction cascade strategy, Ullmann-type coupling along with subsequent C–H activation has been broadly applied in the on-surface synthesis of carbon-based nanomaterials represented by 1D GNRs and 0D nanographene [53,71,72,73].

## 4. Precursors with *Ortho*-Site Substitutions

**Figure 3:**
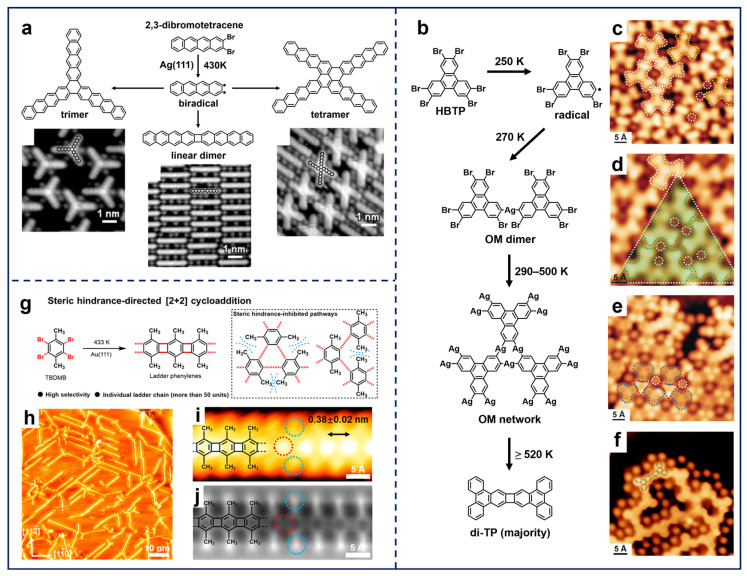
(**a**) Schematic illustration and STM images showing the three reaction products formed by depositing 2,3-dibromotetracene on Ag(111) followed by annealing at 430 K. Reprinted with permission from Ref. [36]. (**b**) Stepwise coupling reaction of the HBTP precursor on Ag(111), including evolution from (**c**) intact HBTP molecules (white) and surface-stabilized monoradicals (yellow), through (**d**) OM dimers (green) and (**e**) OM network, to (**f**) covalent network with dominant di-TP components. Reprinted with permission from Ref. [39]. (**g**) Scheme of the selective [2+2] cycloaddition on Au(111) driven by steric hindrance. (**h**) STM image of the ladder phenylenes formed by depositing the TBDMB precursors on Au(111) held at 433 K. (**i**) STM and (**j**) nc-AFM images of the ladder phenylene chain. Reprinted with permission from Ref. [40].

The above two substituted sites allow the formation of C–C single bonds between phenyl rings, forming 0D, 1D, and extended 2D carbon-based nanostructures in a well-controlled manner. Nevertheless, such a reaction scenario restricts the range of potential nanostructures that can be fabricated. In contrast, molecules with *ortho*-site halogen substitutions enable the formation of ring scaffolds through on-surface Ullmann-type reactions, thereby expanding the reaction protocols for synthesizing π-conjugated structures. Accordingly, several types of ring scaffolds are fabricated and visualized in relevant works [74,75,76,77]. As illustrated in Figure 3a, the study of the 2,3-dibromotetracene molecule on Ag(111) by Meunier et al. demonstrated the typical C–X activation of *ortho*-halogenated molecules in 0D. It, thus, resulted in the formation of a biradical intermediate state, which further evolved into three covalent products, i.e., six-membered-ring trimers via [2+2+2] cycloaddition, four-membered-ring dimers via [2+2] cycloaddition, and double six-membered-ring tetramers via further C–H activation. Notably, the trimer and linear dimer represent the primary products, while the tetramer is relatively minor [36].

Moreover, the *ortho*-site Ullmann-type coupling reaction can be further extended to 2D by the rational design of substituted sites. Kim et al. employed 2,3,6,7,10,11-hexabromotriphenylene (HBTP) molecules as the molecular precursor, consisting of three-fold *ortho*-site halogen substitutions. Based on the hierarchical debromination in response to a gradual annealing strategy, the stepwise Ullmann-type coupling processes on Ag(111) were visualized by trapping the corresponding reaction intermediates (Figure 3b) [39]. A series of STM images resolved the sequential *ortho*-site debromination, which was initiated with the formation of a surface-stabilized monoradical (indicated by the yellow dashed contour in Figure 3c) via the dissociation of only one C–Br bond. Subsequent annealing produced hierarchical structures, including (i) OM dimers with single C–Br bond activation and C–Ag–C coupling at 270 K (Figure 3d), (ii) gradual formation of OM networks with both C–Br bond activation and Ag insertion at 290–500 K (Figure 3e), and (iii) covalent products dominated by four-membered-ring connections via [2+2] cycloaddition at ≥520 K (Figure 3f). Notably, six-membered-ring connection via [2+2+2] cycloaddition was rare, while single-bond connections were also observable. In addition, DFT calculated reaction pathways indicated that the reaction barrier from single-debrominated monomers to OM dimers is lower than the barrier of further debromination into biradical monomers, corresponding to the experimental observation that the single C–Ag–C OM dimers take the dominance after the first annealing step. Moreover, DFT calculations also revealed that the elimination of Ag adatoms from the OM structures was highly endothermic with a high reaction barrier, while the subsequent cycloaddition was highly exothermic with a much lower barrier on Ag(111). The integration of the third diradical to construct [2+2+2] cycloaddition rings was mainly inhibited by the difficulty in gathering with restricted relative positions, leading to the domination of four-membered-ring products rather than six-membered ones. In contrast, in the work by Lin et al., the HBTP molecule showed no significant selectivity between the four- and six-membered rings generated on Au(111) [78]. Furthermore, the OM network formed on Cu(111) via HBTP deposition failed to dissociate Cu adatoms upon further annealing, suggesting that the [2+2] cycloaddition is a highly surface-selective process [38].

Given the diverse reaction pathways of *ortho*-halogenated precursors, extensive research has been conducted on the selective synthesis of the target architectures, as mentioned above. This modulation fundamentally relies on the precursor design and the substrate selection. On the one hand, precursor-driven pathway regulation has emerged as a pivotal strategy. For instance, the incorporation of steric hindrance serves to steer the selectivity of reaction products [79,80]. Liu et al. reported the selective synthesis of ladder phenylene on Au(111) via [2+2] cycloaddition based on 1,2,4,5-tetrabromo-3,6-dimethylbenzene (TBDMB) (Figure 3g,h) [40]. High-resolution STM and nc-AFM images (Figure 3i,j) clearly exhibit the periodicity and the structural configuration of a single 1D chain. Crucially, the two methyl groups on the benzene ring impeded the binding of diradicals through [2+2+2] cycloaddition, thereby directing the formation of the four-membered-ring connection in the covalent chains via [2+2] cycloaddition (Figure 3g).

On the other hand, the selection of substrates is also a crucial factor influencing the on-surface Ullmann-type reactions at the *ortho*-sites, encompassing both types and crystal lattices of metals, as also demonstrated above in the case of HBTP molecules. The template effect introduced by specific crystal planes of substrates, e.g., (111), (110), and (100), has been utilized to regulate molecular adsorption geometries and orientations and assembled structures on surfaces, thereby directing the reaction selectivity [81]. For instance, 2,3-dibromoanthracene molecules formed separated molecular islands of four-membered-ring dimers and six-membered-ring trimers on Au(111). In contrast, only dimers were observed on Au(100) as the molecules adsorbed along the reconstruction rows of the surface, resulting in the high selectivity of [2+2] cycloaddition [82].

Commonly, the products of the same precursor can be diverged because of the differing reactivities of the applied metal substrates. In most cases, on-surface Ullmann-type coupling proceeds without OM intermediates on Au substrates, whereas Ag and Cu surfaces inherently facilitate the formation of OM intermediates during reaction processes. Thus, the coupling reactions on Ag and Cu surfaces proceed through extra steps of the metal insertion, forming OM intermediates with different structures [25,26,37,38,76], which can also effectively guide the reaction pathways. The reaction of 2,3-dibromophenazine molecules on different substrates reported by Chi et al. [83] is a good example. While Au(111) drove the direct [2+2] and [2+2+2] cycloaddition reactions following debromination, a hierarchical evolution of OM intermediates took place on Ag(111) in response to stepwise annealing, leading to the selective formation of [2+2] products.

Therefore, the introduction of *ortho*-site halogen substitution has successfully expanded the synthetic toolbox of π-conjugated nanostructures on surfaces, enabling the construction of ring scaffolds via multiple reaction pathways, including [2+2] and [2+2+2] cycloaddition, as well as further C–H activation [36,37,39]. Critically, such reaction pathways are tunable via dual engineering strategies, including molecular design and substrate selection. As extensively discussed above, steric effects stemming from functional groups on precursors [40], lattice template effects [81,82], and reactivity discrepancies of substrate metals [38,76,83], etc. have collectively established a robust framework for effectively controlling the reaction selectivity.

## 5. Precursors with *Peri*-Region Substitutions

The *peri*-region functionalization between two adjacent benzene rings of the biphenyl structure represents a structurally unique platform for the preparation of GNRs. Among various 1D carbon-based nanostructures, GNRs have attracted considerable attention due to their exceptional electronic properties [4]. In the field of on-surface synthesis, numerous studies have documented the bottom-up synthesis of GNRs with varying topologies. In particular, the pioneering research of Fasel et al. on the synthesis of 7-armchair GNRs (7-AGNRs) on Au(111) in 2010 [71] opened the era of on-surface synthesis and topological engineering of GNRs. Subsequently, a multitude of studies on GNRs have employed similar synthetic methodology, which involves Ullmann-type coupling followed by C–H activation [72,84,85,86,87,88]. In addition to these efforts, AGNRs can also be synthesized via the Ullmann-type coupling alone through the proper design of precursors, which reduces the reaction temperature and enhances the reaction selectivity. The *peri*-region substitution is a notable site of choice, allowing functionalized molecules to undergo cyclization upon on-surface Ullmann-type coupling with the direct formation of six-membered-ring scaffolds [42]. This also represents an effective and stable strategy for the on-surface synthesis of GNR-like structures.

**Figure 4:**
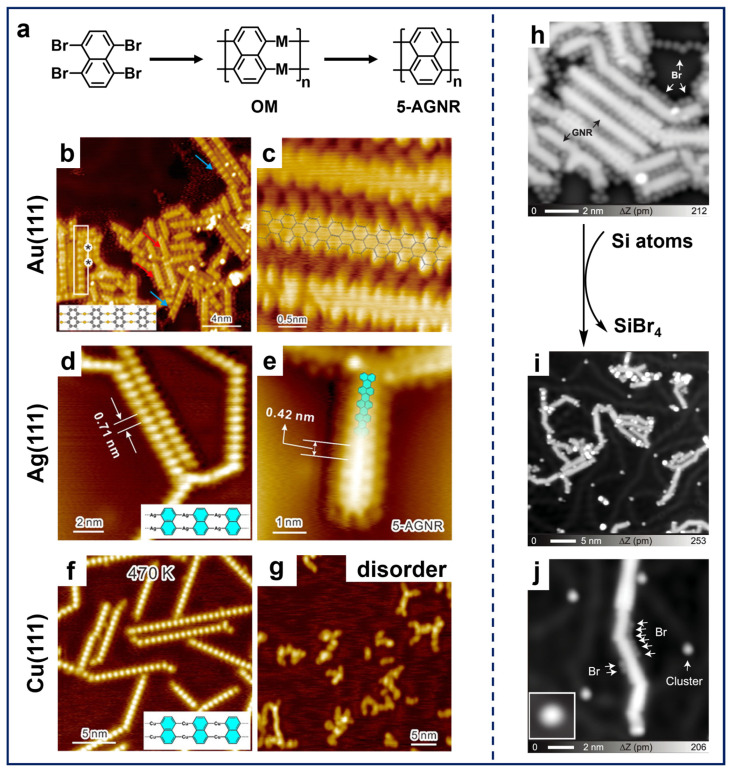
(**a**) Schematic illustration showing on-surface synthesis of 5-AGNRs via Ullmann-type coupling of TBN. (**b**) OM intermediates formed by depositing TBN on Au(111) held at 400 K. The asterisks on the white rectangle indicate the boundaries between the OM chain and the under-reacted molecules. (**c**) 5-AGNRs obtained by subsequent annealing at 470 K. Reprinted with permission from Ref. [41]. (**d**) OM chains obtained after depositing TBN on Ag(111) at RT followed by annealing at 420 K. (**e**) 5-AGNRs fabricated by further annealing at 540 K. (**f**) OM chains formed by RT deposition on Cu(111) and annealing at 470 K. (**g**) Disordered structure obtained after annealing the sample at 600 K. Reprinted with permission from Ref. [89]. (**h**) STM image showing close-packed 5-AGNRs surrounded by Br atoms on Au(111). (**i**) Large-scale and (**j**) close-up STM images showing disperse 5-AGNRs on Au(111) obtained by depositing Si atoms at RT and annealing at 453 K. Reprinted with permission from Ref. [93].

As illustrated in Figure 4a, Chi et al. reported the synthesis of 5-AGNRs by applying the 1,4,5,8-tetrabromonaphthalene (TBN) molecule on different substrates [41,89]. The deposition of TBN molecules on Au(111), Ag(111), and Cu(111) with subsequent annealing resulted in the formation of OM chain structures containing the corresponding metal adatoms (Figure 4b,d,f). Interestingly, further annealing successfully led to the synthesis of 5-AGNRs on Au(111) and Ag(111) (Figure 4c,e), while only disordered structures formed on Cu(111) (Figure 4g). This difference arises because the C–H activation temperature on Cu(111) (~500 K) is significantly lower than the dissociation temperature required for Cu adatoms in the OM chain (~600 K). Apparently, the coupling reactions of TBN molecules demonstrate the ability of *peri*-region precursors in synthesizing nanoribbon structures. These findings also highlight two critical aspects in on-surface synthesis, i.e., the substrate-dependent formation temperatures of OM intermediates and the temperature thresholds for metal adatom removal, providing mechanistic understanding for tailoring the GNRs-related synthesis through substrate engineering.

In addition, the on-surface synthesis of GNRs is influenced by several factors, one of which is the involvement of free halogens in such Ullmann-type reactions. After dehalogenation, halogen atoms are preferentially adsorbed adjacent to organic structures on surfaces, hindering the diffusion of radicals across surfaces. This epitaxial confinement effect influences the progression of polymerization reactions, resulting in the constrained growth of the nanostructure and a decline in structural regularity [60]. Therefore, it is significant to separate halogen atoms from molecular structures, not only for the extended structural growth, but also for the precise property characterization. Among others, a common method is thermal treatment at higher temperatures to induce the desorption of halogen atoms, which may also lead to the simultaneous desorption of oligomer molecules and random activation of C–H bonds [90]. Using atomic hydrogen or molecular hydrogen to convert Br atoms into HBr, which then desorbs from the surface, can also effectively remove Br atoms [37,60,91]. Nevertheless, the high reactivity of hydrogen may interfere with the original Ullmann-type coupling, leading to the quenching of radicals and the formation of byproducts [44,92]. Regarding this aspect, Kawai et al. developed the halogen removal strategy by the introduction of Si atoms [93] into the TBN system. As demonstrated in Figure 4h, a considerable number of Br atoms were adsorbed between the 5-AGNRs fabricated using TBN molecules on Au(111), which resulted in the restricted growth in the GNR’s length. Following the deposition of Si atoms at RT and subsequent annealing, the majority of Br atoms surrounding the GNRs disappeared, resulting in a significant increase in the length of the ribbons (Figure 4i). This phenomenon can be attributed to the combination of Si and Br atoms, which formed the SiBr_x_ (x = 1, 2, 3, 4) clusters and then desorbed, thereby releasing the GNR from the epitaxial confinement. As evidenced by the STM image (Figure 4j), only a residual amount of Br atoms and SiBr_x_ clusters can be discerned. Additionally, another innovative approach to eliminate free halogens from molecular systems is to dose extrinsic sodium (Na), which combines with halogens to form NaX (X represents halogen) salt islands segregated from molecular nanostructures [94]. Thus, the synthesis of nanostructures via on-surface Ullmann-type reactions (especially for those with multiple halogen substituents) is significantly influenced by free halogens as the reduction in heteroatoms not only promotes the seamless growth of target nanostructures but also brings convenience to its characterization.

## 6. Precursors with *Bay*-Region Substitutions

Next, precursors with *bay*-region substitutions and their reaction performances will be mainly discussed. As a site derived from polycyclic aromatic hydrocarbons (PAHs) [95], the *bay*-region has also received significant attention for its ability to prepare carbon-based nanostructures embedded with n-membered rings. Precursors containing *bay*-region halogen substituents serve as building blocks for constructing 2D complicated nanostructures (including polycyclic scaffolds) on surfaces through programmed coupling. These architectures usually originate from synergistic C–X and C–H activation cascades, thereby establishing an alternative paradigm for fabricating π-conjugated systems beyond conventional Ullmann coupling.

**Figure 5:**
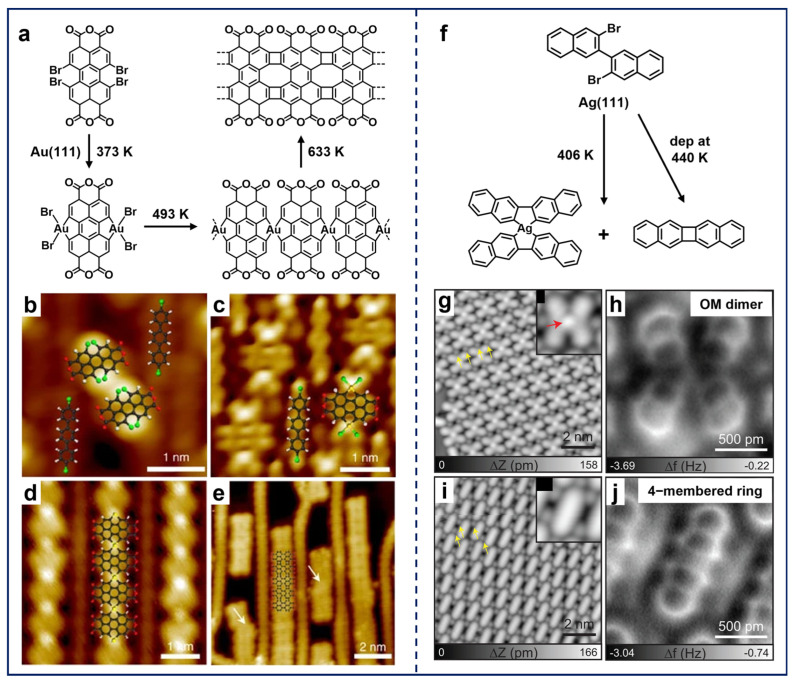
(**a**) On-surface synthesis of graphene-like nanoribbons using Br_4_-PTCDA molecules on Au(111). (**b**) Self-assembly of Br_4_-PTCDA and DBTP molecules after co-deposition at RT. (**c**–**e**) Formation of hierarchical products, including (**c**) OM hybrids formed by annealing at 373 K, (**d**) OM chains obtained after annealing at 493 K, and (**e**) nanoribbons fabricated after annealing at 633 K. Reprinted with permission from Ref. [96]. (**f**) Self-cyclization of DBBN molecules on Ag(111). (**g**,**i**) STM and (**h**,**j**) AFM images of X-shaped OM dimers and self-cyclized monomers obtained after depositing the precursors on Ag(111) and annealing at 406 K. Reprinted with permission from Ref. [44].

The incorporation of non-hexagonal-ring scaffolds into sp^2^-hybridized carbon nanostructures endows them with distinctive physicochemical properties [69]. Zhong et al. embedded four- and eight-membered-ring scaffolds into graphene-like nanoribbons via the Ullmann-type coupling reaction of a *bay*-region substituted precursor, 1,6,7,12-tetrabromo-3,4,9,10-perylene-tetracarboxylic-dianhydride (Br_4_-PTCDA), on Au(111) (Figure 5a) [96]. Notably, the co-deposited 4,4′-dibromo-*p*-terphenyl (DBTP) molecules (Figure 5b) coupled into biphenyl chains after annealing, which further provided the template effect and contributed to the reaction selectivity of Br_4_-PTCDA and the regularity of the products. The reaction sequence was then visualized by STM, involving dehalogenation and subsequent cyclization. Accordingly, the formation of OM hybrids of PTCDA–Au_2_–Br_4_ (Figure 5c) and X-shaped Au-containing OM chains (Figure 5d) was observed. Remarkably, the X-shaped intermediate represents a unique feature of these precursors with *bay*-region substitutions [43,47,49,94]. Finally, the C–H activation occurred along with the removal of Au adatoms, forming the final product of nanoribbons.

While the Br_4_-PTCDA molecule represents a standard *bay*-region precursor, some non-canonical molecules with the potential to transform into *bay*-region forms have also been extensively applied in the on-surface preparation of non-hexagonal-ring scaffolds. In the case of single-sided *bay*-region substitution, although the *trans*-form is generally more stable than the corresponding *cis*-form, the rotational freedom of the terminal groups around C–C single bonds leads to the equivalent conformational alignment. Consequently, the reaction also follows the sequence from OM intermediates to covalent structures [49,97]. For instance, Kawai et al. pioneeringly adopted the 3,3′-dibromo-2,2′-binaphthalene (DBBN) molecules for the on-surface synthesis of radialene structures (Figure 5f) [44]. After the deposition of DBBN molecules on Ag(111) and annealing at 406 K, the X-shaped OM dimers (Figure 5g,h) and self-cyclized monomers (Figure 5i,j) were obtained. Moreover, the cyclized monomer can also be selectively obtained via deposition on the substrate kept at 440 K. Thus, this work presents an alternative reaction pathway for *bay*-region precursors, namely self-cyclization to form a four-membered-ring scaffold.

**Figure 6:**
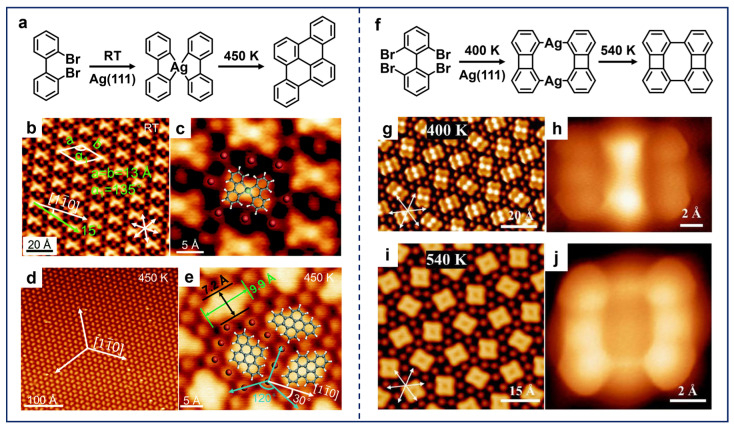
(**a**) Dimerization of DBBP molecules on Ag(111). (**b**) Large-scale and (**c**) close-up STM images of OM dimers obtained after the deposition of DBBP molecules on Ag(111) at RT. (**d**,**e**) Rhombic molecules obtained after annealing the sample at 450 K. Reprinted with permission from Ref. [46]. (**f**) Dimerization of TBBP molecules on Ag(111). (**g**,**h**) OM dimers obtained after depositing TBBP molecules on Ag(111) at RT and annealing at 400 K. (**i**,**j**) Biphenylene dimers obtained after annealing the sample at 540 K. Reprinted with permission from Ref. [48].

In addition, the potential *bay*-region precursors also allow for the on-surface synthesis of a more diverse range of nanostructures as the additional incorporation of C–H activation supplements the self-cyclization pathway to facilitate the construction of polycyclic structures. A typical example is the work by Zhu et al., which reported the dimerization of 2,2′-dibromo-biphenyl (DBBP) molecules on Ag(111), leading to the construction of naphthalene scaffolds (Figure 6a) [46]. Upon RT deposition, the X-shaped OM dimers were formed (Figure 6b,c), which is similar to the case shown in Figure 5g,h. Subsequent annealing led to the formation of the double six-membered-ring scaffolds, accompanied by the adjacent C–H activation (Figure 6d,e). More recently, based on the same molecular precursor (DBBP) adsorbed on Cu(111), photolytic activation was employed as an alternative activation method, leading to the selective fabrication of dimers with eight-membered-ring connections directly through Ullmann-type coupling [98]. This is in contrast to the case of thermal activation, which yielded a variety of coupled reaction products.

Comparatively, in the case of precursors with double-sided *bay*-region substituents, the resulting product exhibits a remarkably distinct structure. As an extension, Zhu et al. further explored the coupling of 2,2′,6,6′-tetrabromo-1,1′-biphenyl (TBBP) molecules on Ag(111) (Figure 6f) [48]. STM images and DFT calculations validated that one of the *bay*-region sites on the TBBP molecule underwent self-cyclization, while Ag adatoms were inserted into the other site after annealing at 400 K, forming OM dimers (Figure 6g,h). Further annealing at 540 K then produced covalent dimers containing four- and eight-membered-ring scaffolds (Figure 6i,j). In contrast, calculations for the DBBP molecule demonstrate that it is stabilized on the surface by the formation of X-shaped OM dimers. The energy barrier for the subsequent occurrence of Ullman coupling is lower than that for self-cyclization. This results in a reaction selectivity that is distinct from that of TBBP on Ag(111). Therefore, these two cases shown in Figure 6 intuitively indicate that the subtle modification of substituted sites would eventually lead to completely different reaction products, providing atomic-level insight into the important role of functionalization.

Notably, the Ullmann-type coupling of *bay*-region precursors displays several distinctive characteristics in comparison with others, including the presence of X-shaped OM intermediates [43,44], the formation of various polycyclic structures [96], etc. In addition, some molecules can be transformed into *bay*-region precursors by adjusting their configuration [46,47,48,97], thus affording a greater array of options to the design of precursors. Importantly, this kind of reaction further enhances structural diversity in the synthesis of low-dimensional π-conjugated nanomaterials on surfaces.

## 7. Precursors with Hybrid Substituted Sites

The reactions of precursors with singly substituted sites have been systematically summarized above. To further extend the scope of on-surface Ullmann-type reactions and the variety of resulting nanostructures, precursors with hybrid substituted sites have also been adopted. The corresponding reactions may also enable hierarchical reaction engineering by virtue of site-specific reactivity gradients. Despite these potential advantages, the employment of precursors with hybrid substituted sites also introduces a greater number of halogen substituents to molecular systems. At the same time, this may result in a more complex reaction scenario of the molecules with approximate reaction selectivity at various sites, leading to the formation of more by-products, more free halogen atoms, and mixed nanostructures. Considering these difficulties, the design of multi-site precursors presents significant challenges. Firstly, the selection of substituted sites must prioritize high reaction selectivity. Secondly, the reaction process must be structured hierarchically. Finally, the treatment of impurities, such as free halogens, should also be considered. These conditions facilitate the stepwise evolution of intermediates while simultaneously suppressing by-product formation through the prevention of uncontrolled cross-coupling between different sites.

**Figure 7:**
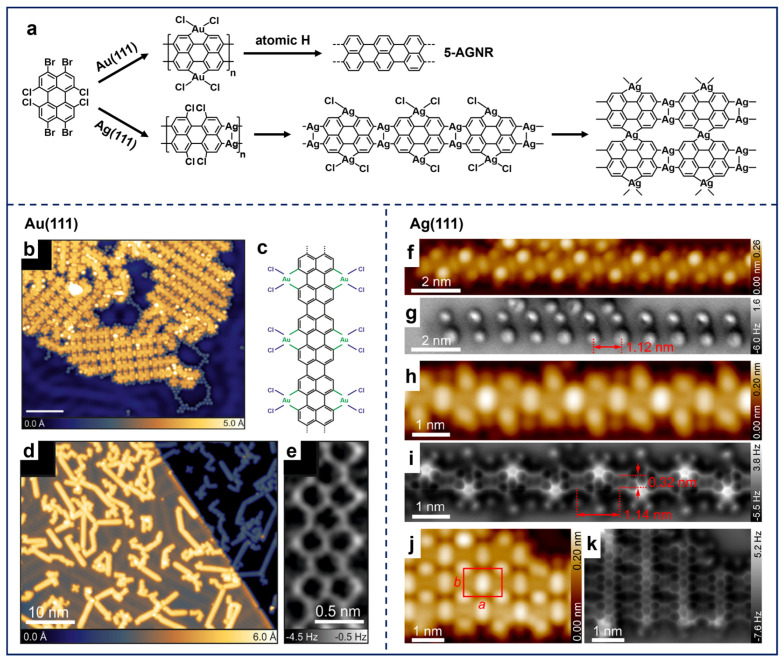
(**a**) Polymerization processes of TBTCP molecules on Au(111) and Ag(111). (**b**) STM image of GNROHs obtained by depositing TBTCP on Au(111) and annealing at 433–463 K. (**c**) Chemical structure of GNROH. (**d**) 5-AGNRs obtained after dosing atomic H on the surface held at 373 K. (**e**) Nc-AFM image showing the aromatic backbone of 5-AGNR. Reprinted with permission from Ref. [100]. (**f**–**k**) STM and nc-AFM images of all the OM structures formed by depositing TBTCP on Ag(111) and subsequent annealing, including (**f**,**g**) chlorinated silver–organic nanoribbon (420 K), (**h**,**i**) GNROH (550 K), and (**j**,**k**) 2D silver–organic frameworks (660 K). Reprinted with permission from Ref. [101].

To the best of our knowledge, there are only a few studies that have successfully incorporated hybrid substituted sites into molecular precursors and demonstrated hierarchical reaction processes. For precursors with the combination of *para*-, *meta*-, and *ortho*-sites, the selectivity of the sequential dehalogenation of such hybrid bromine-substituted benzene molecules [99] has been visualized based on STM observations, ending with the formation of OM structures. In the case of selecting molecules with divergent substituted sites and halogen substituents with discrete dehalogenation temperatures, Godlewski et al. reported the polymerization of 3,4,9,10-tetrabromo-1,6,7,12-tetrachloroperylene (TBTCP) molecules on Au(111), featuring both a Br-substituted *peri*-region and a Cl-substituted *bay*-region (the top in Figure 7a) [100]. Following the deposition of TBTCP molecules at RT and annealing, the coupling reaction initiated at the *peri*-region, while Au atoms were inserted into the *bay*-region, resulting in the formation of the graphene-nanoribbon OM hybrids (GNROHs) (Figure 7b,c). Subsequently, the synthesis of 5-AGNRs was successfully achieved by removing Cl and Br atoms from the GNROHs and underlying surface through the aforementioned method of dosing atomic H (Figure 7d,e) [60].

In addition to the above illustration of synthesizing AGNRs on Au(111), this multi-substituted precursor also displayed sequential reaction processes on Ag(111). It is worth noting that Chi et al. successfully expanded the 1D GNROHs to 2D silver–organic networks using the same TBTCP molecules on Ag(111) (the bottom in Figure 7a) [101]. The RT deposition of TBTCP molecules and stepwise annealing resulted in the formation of hierarchical OM nanostructures. Structural evolution progressed systematically from silver–organic nanoribbons at 420 K (Figure 7f,g), through further Ag-embedded nanoribbon configurations at 550 K (Figure 7h,i), ultimately forming 2D silver–organic networks at 660 K (Figure 7j,k). This hierarchical evolution was governed by the site-specific Ag adatom insertion, with the initial insertion occurring preferentially at *peri*-regions, followed by the subsequent involvement of *bay*-regions, demonstrating an ordered substitution hierarchy during dehalogenation. DFT calculations further revealed that the distinct product formation on Ag(111) versus Au(111) stems from a fundamental difference in bond dissociation energetics, i.e., the significantly lower activation barrier for Ag–Cl bond cleavage compared to Au–Cl bonds enables kinetically favorable fusion between OM nanoribbons on Ag(111).

## 8. Conclusions

In conclusion, we summarize recent research progress in the synthesis of low-dimensional carbon-based nanostructures based on halogen-substituted molecules featuring multiple substituted sites, including *para*-, *meta*-, and *ortho*-sites, *peri*- and *bay*-regions, and their combination. The reaction processes of these different sites and their representative works are also discussed. Conventional approaches utilizing *para*- and *meta*-sites focus on single C–C bond formation, serving as foundations for constructing 1D linear chains and 2D extended networks, respectively. Expanding beyond these conventional strategies, *ortho*-site substitutions introduce angular connectivity that diversifies structural outcomes, enabling precise fabrication of both four- and six-membered-ring scaffolds. In addition, *peri*-region precursors exhibit geometric selectivity that specifically directs the formation of six-membered-ring scaffolds. At the frontier of complexity, *bay*-region coupling strategies often integrate Ullmann-type coupling with C–H activation processes, thereby broadening synthetic horizons to prepare both multi-membered-ring scaffolds and interconnected polycyclic junctions. These on-surface Ullmann-type coupling reactions discussed herein also demonstrate how site-specific substitution governs structural evolution across dimensional and topological aspects.

In addition, an on-surface Ullmann-type reaction has been well established as a versatile synthetic approach that is readily activated under surface-mediated conditions and enables precise engineering of carbon-based nanostructures with targeted connectivity. Simultaneously, advanced characterization techniques such as UHV-STM and nc-AFM provide atomic-scale resolution to visualize reaction intermediates and validate product architectures. While extensive studies have been focused on molecular precursors with single substituted sites, studies leveraging multi-site hybrid substitution patterns remain comparatively underexplored, which hold significant potential for advancing structural complexity and diversity. In addition, strategic incorporation of mixed halogen species offers an additional dimension for designing stepwise reaction cascades as site-specific dehalogenation kinetics can be exploited to control hierarchical reaction processes. These aspects present a considerable opportunity for fully exploiting the programmable feature of Ullmann-type reactions. Concurrently, a more comprehensive study on the modulation of reactions [102] is also required to construct large-scale nanostructures with low defect density, which should be significant for the fabrication of next-generation molecule-based electronic devices. Moreover, adopting relatively inert surfaces (e.g., insulating or semiconducting substrates), which possess lower catalytic activity, remains under-explored for on-surface Ullmann-type reactions [77], yet should be overcome for future practical molecular-based electronic applications. Ultimately, while most current studies operate on metal substrates under UHV environments, translating these surface-confined syntheses into functional electronic devices remains both a fundamental scientific challenge and a pivotal technological frontier, requiring interdisciplinary innovation at the interface of surface chemistry and materials engineering.
